# Mitochondrial DNA in extracellular vesicles declines with age

**DOI:** 10.1111/acel.13283

**Published:** 2020-12-23

**Authors:** Stephanie Lazo, Nicole Noren Hooten, Jamal Green, Erez Eitan, Nicolle A. Mode, Qing‐Rong Liu, Alan B. Zonderman, Ngozi Ezike, Mark P. Mattson, Paritosh Ghosh, Michele K. Evans

**Affiliations:** ^1^ Laboratory of Epidemiology and Population Science National Institute on Aging National Institutes of Health Baltimore MD USA; ^2^ Laboratory of Neuroscience National Institute on Aging National Institutes of Health Baltimore MD USA; ^3^ Laboratory of Clinical Investigation, National Institute on Aging National Institutes of Health Baltimore MD USA; ^4^ Present address: Perelman School of Medicine University of Pennsylvania Philadelphia PA USA; ^5^ Present address: NeuroDex Natick MA USA

**Keywords:** aging, biomarker, circulating cell‐free mitochondrial DNA, exosomes, extracellular vesicles, intercellular communication, microvesicles, mitochondrial DNA

## Abstract

The mitochondrial free radical theory of aging suggests that accumulating oxidative damage to mitochondria and mitochondrial DNA (mtDNA) plays a central role in aging. Circulating cell‐free mtDNA (ccf‐mtDNA) isolated from blood may be a biomarker of disease. Extracellular vesicles (EVs) are small (30–400 nm), lipid‐bound vesicles capable of shuttling proteins, nucleic acids, and lipids as part of intercellular communication systems. Here, we report that a portion of ccf‐mtDNA in plasma is encapsulated in EVs. To address whether EV mtDNA levels change with human age, we analyzed mtDNA in EVs from individuals aged 30–64 years cross‐sectionally and longitudinally. EV mtDNA levels decreased with age. Furthermore, the maximal mitochondrial respiration of cultured cells was differentially affected by EVs from old and young donors. Our results suggest that plasma mtDNA is present in EVs, that the level of EV‐derived mtDNA is associated with age, and that EVs affect mitochondrial energetics in an EV age‐dependent manner.

## Graphical abstract

Mitochondrial dysfunction plays a central role in aging. A portion of circulating cell‐free mitochondrial DNA (mtDNA) isolated from blood is present in extracellular vesicles (EVs), which are small, lipid‐bound vesicles that shuttle macromolecules as part of intercellular communication systems. The level of EV‐derived mtDNA declines with age, and these EVs affect mitochondrial energetics in an EV age‐dependent manner.

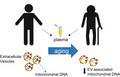

## 1 INTRODUCTION

Aging is a complex systematic process, which is associated with the progressive loss of organ and tissue function over the lifetime resulting in an increased risk of various age‐associated diseases and mortality. The contribution of mitochondrial dysfunction to aging was initially theorized over five decades ago in the mitochondrial free radical theory of aging (Harman, 1965). Since then, accumulating evidence suggests that a decline in mitochondrial function is an important contributor to the aging process and also for driving age‐related disease (Kujoth et al., 2005; Lopez‐Otin et al., 2013; Park & Larsson, 2011; Ross et al., 2013; Trifunovic et al., 2004; Vermulst et al., 2008).

The mitochondrial genome encodes 37 genes including 13 proteins, which are vital for mitochondrial oxidative phosphorylation and cellular energetics. Mitochondrial DNA (mtDNA) mutations drive premature aging in mouse models (Kujoth et al., 2005; Park & Larsson, 2011; Ross et al., 2013) and recently have been shown to increase with human age (Zhang et al., 2017). mtDNA copy number (the number of mtDNA molecules per cell) was also found to decline with human age in peripheral blood mononuclear cells (Mengel‐From et al., 2014; Zhang et al., 2017).

Cellular mtDNA can be released outside of the cell as circulating cell‐free mtDNA (ccf‐mtDNA). Ccf‐mtDNA can act as a damage associated molecular pattern (DAMP) molecule leading to activation of the innate immune response following cellular damage or stress (Mills et al., 2017; West et al., 2011; Zhang et al., 2010). Ccf‐mtDNA can be reliably measured from blood plasma and serum making it attractive for biomarker development (Boyapati et al., 2017). Elevated ccf‐mtDNA levels are associated with inflammatory diseases and cancer, as well as with trauma or tissue injury, including myocardial infarction and sepsis (Boyapati et al., 2017; Krysko et al., 2011; Schwarzenbach et al., 2011). A recent European study has shown a slight decline in ccf‐mtDNA comparing levels in children to middle‐aged individuals followed by a gradual increase in ccf‐mtDNA in the elderly (Pinti et al., 2014). These emerging data suggest that ccf‐mtDNA may indicate and/or contribute to various physiological and pathological conditions.

Ccf‐mtDNA has been reported to be present in extracellular vesicles (EVs). EVs are small lipid membrane vesicles of ~30–400 nm that are released from cells. Extracellular vesicle is a general term encompassing several types of vesicles including exosomes, microvesicles and apoptotic bodies. The biogenesis pathway is different for each of these EVs, but current isolation techniques make it difficult to distinguish the different subtypes. Exosomes are released through the fusion of the multivesicular body to the plasma membrane. Microvesicles are formed through pinching off of the plasma membrane and apoptotic bodies are released during apoptosis (Verderio et al., 2014; Yanez‐Mo et al., 2015). EVs also contain proteins, lipids, and nucleic acids that can be delivered to target cells (Elzanowska et al., 2020; Greening et al., 2017; Kim et al., 2017). Recent data indicate that ccf‐mtDNA can be detected in EVs derived from myoblasts, astrocytes and glioblastoma cells grown *in vitro* and also in plasma EVs from women with hormonal therapy‐resistant breast cancer (Guescini et al., 2010a; Guescini, Guidolin, et al., 2010; Sansone et al., 2017). Mitochondrial markers in larger platelet‐derived EVs have been detected using flow cytometry (Marcoux et al., 2019) and visualized within EVs in electron microscopy images (Phinney et al., 2015; Puhm et al., 2019).

EVs have been shown to be important mediators in intercellular communication (Mathieu et al., 2019; van Niel et al., 2018; Yanez‐Mo et al., 2015). In fact, in monocytes, mitochondria containing EVs were important for stimulating Type I IFN and TNF responses in endothelial cells (Puhm et al., 2019). Transfer of mitochondrial components using EVs has been implicated in hormonal therapy‐resistant breast cancer (Sansone et al., 2017), and in mesenchymal stem cell management of oxidative stress (Phinney et al., 2015). Recently, it was also demonstrated that human plasma contains intact cell‐free mitochondria (Dache et al., 2020). These data indicate that mitochondrial components may be important functional cargo of EVs in specific cellular contexts, but little is known about whether this is a broad mechanism for EVs. Furthermore, little is known about whether mitochondrial components are contained within EVs under normal physiological conditions or only in response to an injury, stress, or specific disease state.

Previously, we reported that EV concentration declined with age in a cross‐sectional and longitudinal study (Eitan et al., 2017). EVs isolated from older individuals were preferentially internalized by B cells compared to EVs from younger individuals. These data indicate that EVs from older individuals may contain different cargo than EVs from younger individuals. In the current study, we examine whether mtDNA can be detected in human plasma EVs and whether mtDNA levels are altered with human age.

## 2 RESULTS

### 2.1 Plasma EVs contain mtDNA

**Figure 1:**
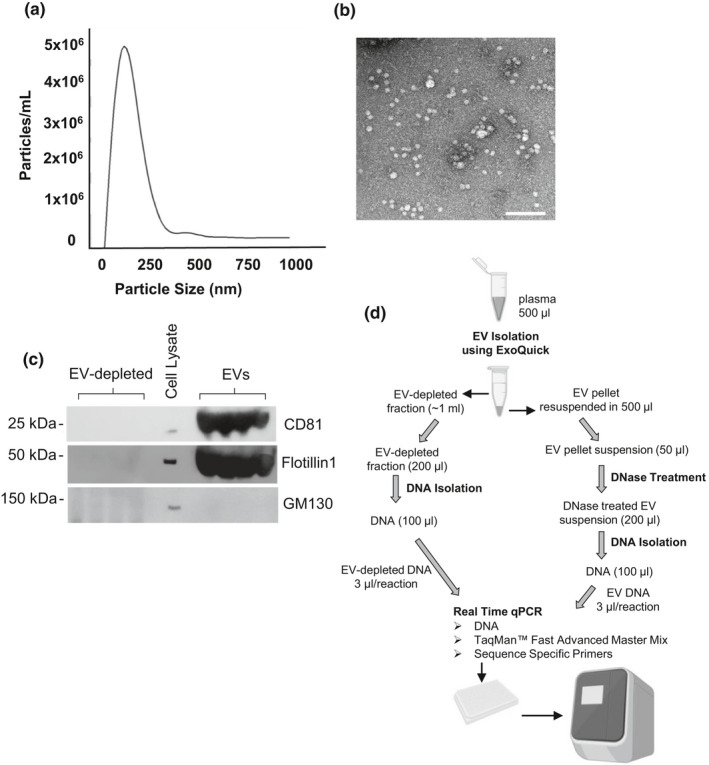
Characterization of EVs and schematic of workflow. (a) Two sets of four plasma samples were pooled and divided into four equal aliquots, EVs were isolated and analyzed by Nanoparticle Tracking Analysis. Coefficient of variation for each experiment (*N* = 4) was 6.8% and 2.0%, respectfully. (b) Electron microscopy showed EVs of typical size and morphology (scale bar = 200 nm). (c) EV markers are enriched in EVs. We used the same EVs that were isolated and measured in part A. The EVs were lysed and EVs, and EV‐depleted fractions and HeLa cell lysate were analyzed by SDS‐PAGE and immunoblotted with antibodies against the indicated EV markers. GM130 was used as a negative control. (d) Flow chart of experimental design. For the analysis of EV mtDNA levels with age, 0.45 ml of plasma was used

Most studies have examined mtDNA in plasma without analyzing whether circulating mtDNA is present in EVs as well as the EV free fraction. Here, we wanted to examine whether mtDNA was present in the cell‐free circulating state as well as encapsulated within plasma EVs. To do this, we pooled plasma from 4 different individuals and had two independent plasma pools. From this pooled plasma, we aliquoted the pools into 4 different samples. We isolated EVs from each sample and kept the EV‐depleted fraction. Isolated plasma EVs were validated according to the International Society of Extracellular Vesicles guidelines (Thery et al., 2018). Size distribution of the isolated vesicles was confirmed by nanoparticle tracking analysis (NTA) with a peak around 150 nm (Figure 1a). The electron microscopy image showed intact, cup‐shaped vesicles in the expected morphology of EVs (Figure 1b). Established EV markers were present in the EV samples and absent in the EV‐depleted supernatant (Figure 1c). This confirms the size, morphology, and protein markers that are characteristic of EVs.

**Figure 2:**
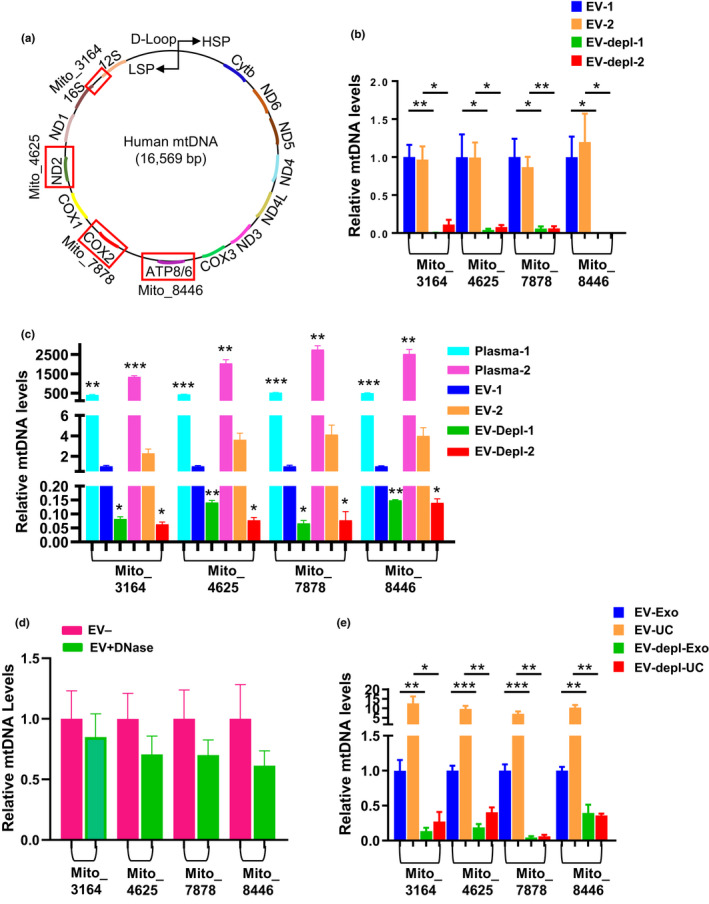
mtDNA is present in EVs (a) Schematic of mtDNA primer design. mtDNA primer regions are boxed in red and bolded next to each boxed region is the primer name and the starting nucleotide. Mito_3164 primer crosses the 16S rRNA and tRNA‐Ile1 regions and the forward sequence starts at bp 3164. Mito_4625 targets the ND2 region of the genome starting at bp 4625. Mito_7878 targets the COX2 region of the genome starting with bp 7878. Mito_8446 targets the ATP8 gene and begins at bp 8446. (b) Two sets of pooled plasma samples (from *n* = 4 individuals) were divided into four equal aliquots. EVs were isolated, and both EV and EV‐depleted fractions were used for DNA isolations. Four different primers were used to analyze mtDNA content in DNA derived from EV and EV‐depleted fractions using qPCR. (c) Plasma was pooled from the same individuals as in (b) and DNA was isolated from plasma, EVs or EV‐depleted fractions (*n* = 3 per condition). mtDNA was analyzed as above. (d) Plasma EVs were isolated with or without the DNase treatment step (*n* = 4 per condition). (e) Pooled plasma samples were divided into 8 equal aliquots. EVs were isolated for half of the aliquots using ultracentrifugation (UC) and the other half using ExoQuick™ (Exo). The EV and EV‐depleted (EV‐depl) fractions from each isolation method were used for DNA isolations for each histogram, and mtDNA levels were normalized to the EV group for each different primer set. Histograms represent the mean ± SEM. *p < 0.05, **p < 0.01 and ***p < 0.001 by Student *t* test

For each EV sample and EV‐depleted sample, we then isolated the DNA. A flow chart showing the experimental workflow is shown in Figure 1d. During the DNA isolation from EVs, we DNase treated to degrade any DNA material on the outside of the EVs or that was associated with the EVs (Figure 1d). We then performed quantitative real‐time PCR (qPCR) analysis using mitochondrial genome specific primers that were designed against four different regions of the mitochondrial genome (Figure 2a, Supporting Information Table S1). The primers were designed to hybridize to the junction between 16S rRNA (*MT*‐*RNR2*) and tRNA‐Ile1 (*MT*‐*TL1*) genes (Mito_3164), the NADH dehydrogenase 2 (*MT*‐*ND2*) gene region (Mito_4625), the ATP8 (*MT*‐*ATP8*) gene region (Mito_8446), and the Cytochrome c oxidase subunit 2 (*COX2*) gene region (Mito_7878) (Figure 2a). All primer sets were verified using BLAST (http://www.ncbi.nlm.nih.gov Pearson & Lipman, 1988) only to align with the mitochondrial genome (Malik et al., 2011). We used each of these four different primers to amplify different regions of the mitochondrial genome in our EV and EV‐depleted samples. The associated amplification plots for mtDNA isolated from EVs are shown in Supporting Information Figure S1A. Furthermore, the qPCR products were visualized by electrophoresis in SYBR‐safe stained gels and a single amplification product was observed at the expected size (Figure S1B,C). We found that the circulating mtDNA was significantly enriched in the EVs compared to the EV‐depleted fractions (Figure 2b). Similar results were obtained with all four mitochondrial primer sets.

We then wanted to further compare the levels of mtDNA in plasma with that found in EVs and the EV‐depleted fraction within plasma. To test this, we took pooled plasma from the same individuals as above and isolated DNA from plasma, EVs, and the EV‐depleted fraction. Interestingly, the highest levels of mtDNA were detected in plasma compared to EVs and EV‐depleted fraction (Figure 2c). To address whether mtDNA may be on the outside of EVs or co‐precipitated during the EV isolation procedure, we isolated EVs from plasma and either DNase treated, or mock treated the EVs prior to isolating the DNA. We then performed qPCR using the mtDNA primer sets. This analysis found that there was more mtDNA in the mock treated samples compared to DNase treated samples; however, these differences were not significant (Figure 2d). Therefore, this suggests that in plasma mtDNA can be contained in EVs, which makes this pool of mtDNA resistant to DNase treatment.

We also further validated our results using another EV isolation method. We chose to use ultracentrifugation since it is a widely used method for EV isolation (Gardiner et al., 2016). Pooled plasma samples were isolated using either EV isolation method and DNA was isolated from the EV and EV‐depleted fractions. We found that ccf‐mtDNA was also significantly enriched in the EVs isolated using ultracentrifugation compared to the EV‐depleted fraction (Figure 2e). These data suggest that ccf‐mtDNA in plasma can be encapsulated in EVs.

### 2.2 Significant correlation between EV mtDNA levels amplified with different primers

To address whether mtDNA levels in EVs are altered with human age, we chose a subcohort of individuals across the lifespan from the Healthy Aging in Neighborhoods of Diversity Across the Life Span (HANDLS) study. The cohort is made up of community‐dwelling individuals from three different age groups: young, middle aged, and old (Table 1). We had previously isolated and examined the EV characteristics of this cohort (Eitan et al., 2017). Although mtDNA levels were higher in EVs isolated using ultracentrifugation (Figure 2c), the coefficient of variance for this technique (22%) was higher than using precipitation methods (9%) (Figure 2e). Furthermore, we previously reported that precipitation methods were more reproducible then using ultracentrifugation and allowed for the processing of a large number of clinical samples (Eitan et al., 2017). Here, we utilized the methodology described above to analyze the EV mtDNA levels.

**Table 1:** Demographics for EV mtDNA and aging cohort

Characteristics	Visit	Young	Middle	Old	*p* value
*N*	1	21	28	6	
	2	25	29	13	
Age mean (SD)	1	32.1 (1.46)	47.5 (2.76)	59.8 (3.02)	0.001
	2	37.0 (1.70)	52.0 (2.53)	66.0 (2.24)	0.001
Male *n* (%)	1	12 (57%)	17 (61%)	1 (17%)	0.138
	2	16 (64%)	17 (59%)	5 (38%)	0.309
AA *n* (%)	1	10 (48%)	14 (50%)	3 (50%)	0.985
	2	11 (44%)	15 (52%)	9 (69%)	0.335
BMI mean (*SD*)	1	25.1 (5.32)	25.6 (4.94)	30.6 (5.82)	0.072
	2	27.1 (5.96)	26.0 (5.26)	28.1 (4.87)	0.509

Age and BMI are reported as mean ± SD. Pearson's chi‐squared tests were used to analyze differences among the age groups for sex and race (African American, AA). One‐way ANOVAs were used to analyze differences among the age groups for BMI and age.

**Figure 3:**
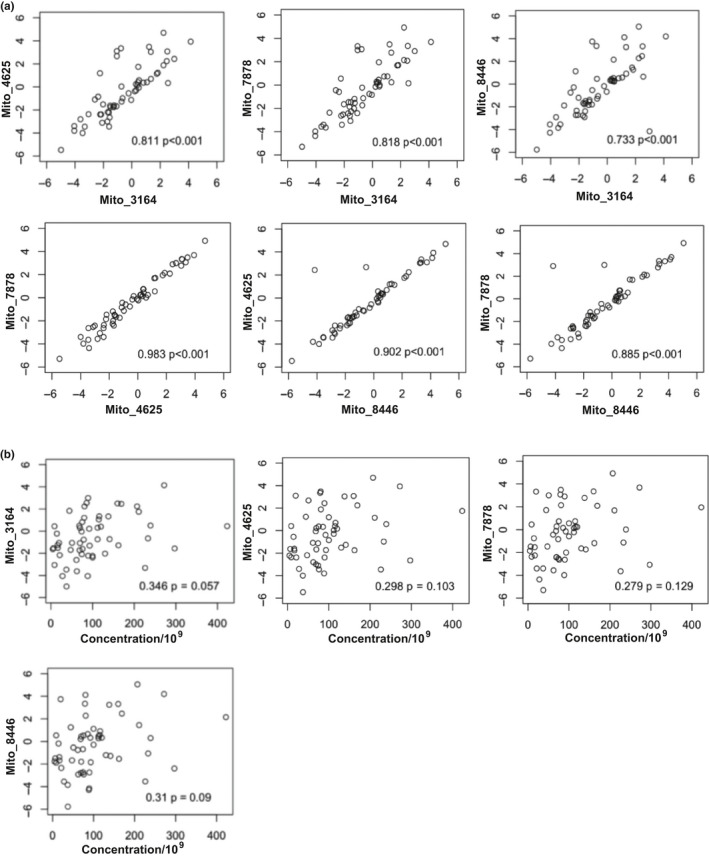
mtDNA primers positively correlate with each other but not with EV concentration (a) EVs were isolated from plasma from 55 individuals across different age groups at visit 1. DNA was isolated and circulating cell‐free mtDNA levels were measured using mtDNA specific primers from four different regions of the mitochondrial genome via qPCR. Correlations between primers were assessed by Pearson correlation, and r values and *p* values are indicated. mtDNA levels were log_2_ transformed. Similar results were obtained for visit 2 (Figure S2). (b) EV concentration was obtained using Nanoparticle Tracking Analysis and the relationship between EV concentration and EV mtDNA levels (log_2_ transformed) were analyzed by Pearson correlation. *r* values and *p* values are indicated. Similar results were obtained for visit 2 (Figure S2)

We isolated DNA from the EVs and used four different mtDNA‐specific primer sets for qPCR analysis. First, we analyzed the relationship between the mtDNA levels amplified using each of the different primer sets in this cohort. We found that mtDNA levels amplified with each primer set were significantly positively correlated with the other primer sets using Pearson correlation (Figure 3a). Similar results were obtained from visit 1 (Figure 3) and visit 2 (Figure S2a). Thus, we can detect and amplify different regions of the mitochondrial genome from human plasma EVs and each primer set is highly correlated with the other primer sets.

### 2.3 EV mtDNA levels are not associated with EV concentration

Previously, we found that EV concentration decreases with age (Eitan et al., 2017). To exclude the possibility that changes in mtDNA abundance with age were not simply due to changes in EV concentration (Eitan et al., 2017), we examined the relationship between EV concentration and mtDNA levels. We found no significant association between EV concentration and mtDNA levels for each of the four primer sets (Figure 3b). Similar results were obtained from visit 1 (Figure 3) and visit 2 (Figure S2a).

### 2.4 Plasma EV mtDNA content declines with age

To assess whether there are differences in plasma EV mtDNA across the lifespan, we examined mtDNA levels in relation to age using linear mixed model regression. This cohort contains individuals that donated plasma at two different time points approximately five years apart. This allows us to analyze cross‐sectional changes in mtDNA levels at both visit 1 and visit 2 and examine longitudinal changes as well.

**Figure 4:**
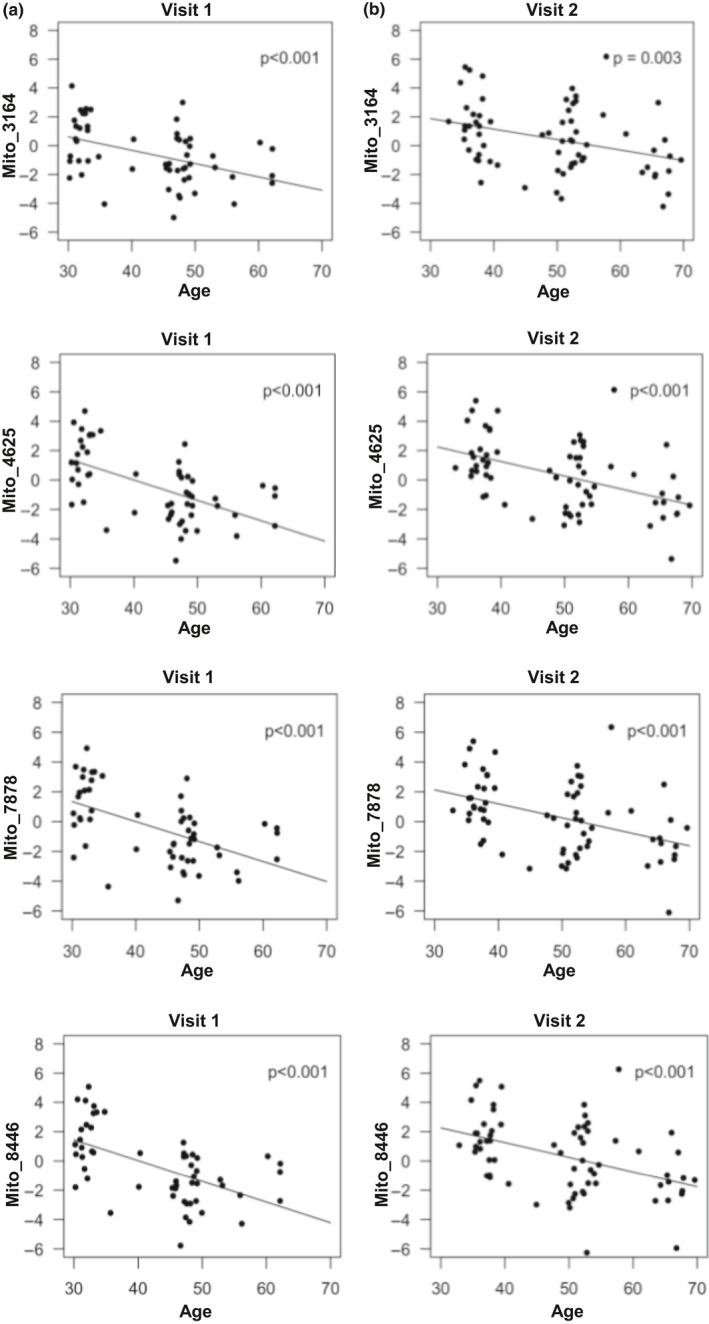
Plasma EV mtDNA levels decrease with age. EVs were isolated from the plasma of 55 individuals at visit 1, and 67 individuals at visit 2 of an aging subcohort from the HANDLS study, outlined in Table 1. DNA was purified and mtDNA was amplified using mitochondrial genome specific primers via qPCR. Linear mixed model regression was used to analyze the relationships in a cross‐sectional analysis at the two different time points. Visit 1 (a) and Visit 2 (b), demonstrate the relationship between EV mtDNA levels (log_2_ transformed) and age at two time points, approximately 5 years apart. *p* Values are indicated

We observed age‐associated differences in plasma EV mtDNA levels. Plasma EV mtDNA was significantly and negatively associated with age in our cross‐sectional analyses at both visit 1 (Figure 4 panel a) and visit 2 (Figure 4 panel b). In addition, this association was observed for each of the four primer pairs tested (Figure 4).

**Figure 5:**
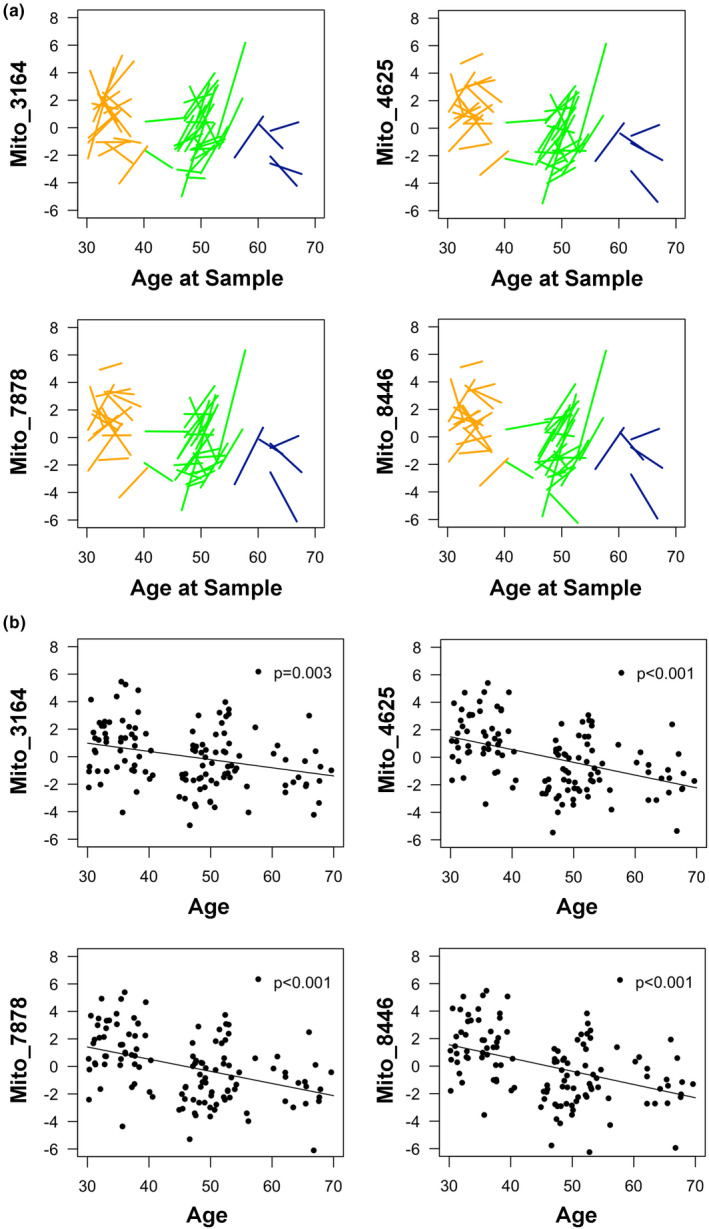
Plasma EV mtDNA levels decrease with age longitudinally. EVs were isolated from the plasma of 55 individuals at visit 1, and 67 individuals at visit 2 of an aging subcohort from the HANDLS study, outlined in Table 1. DNA was purified and mtDNA was amplified using mitochondrial genome specific primers via qPCR. (a) Spaghetti plots show longitudinal changes in EV mtDNA levels (log_2_ transformed). The start of each line represents the starting EV mtDNA level and the end of the line represents the EV mtDNA level at the last time point. (b) EV mtDNA levels significantly decreased over time between measurements. Dots represent the mtDNA level (log_2_ transformed) per individual at each sample collection. *p* values are in the upper right‐hand corner of each figure. Linear mixed model regression was utilized to model the EV mtDNA levels over time, for the cohort matched by sex and race

To visualize the longitudinal changes in EV mtDNA levels, we used spaghetti plots where each individual's mtDNA level over time can be visualized (Figure 5a). Each line starts at the mtDNA level and age at visit 1 and ends at the mtDNA level and age at visit 2. To test whether there were longitudinal changes in EV mtDNA levels, we used linear mixed model regression. This analysis showed that EV mtDNA levels significantly decreased longitudinally from visit 1 to visit 2 (Figure 5b). Significant decreases were observed for all four mitochondrial genome primer sets (Figure 5b).

### 2.5 EVs from young and old individuals affect mitochondrial function

Given that EVs from older individuals contain significantly lower levels of mtDNA and that EVs are important mediators in intercellular communication, we wanted to examine whether EVs from young and old individuals have different functional consequences in vitro on mitochondrial energetics. First, we added different amounts of plasma EVs to HeLa cells and measured oxygen consumption rates (OCR) using Agilent Seahorse technologies. We observed an EV concentration dependent response in maximal respiration to increasing EV doses (Figure S3). Therefore, we chose the lower dose (8.1E4) of EVs to use in our experiments.

**Figure 6:**
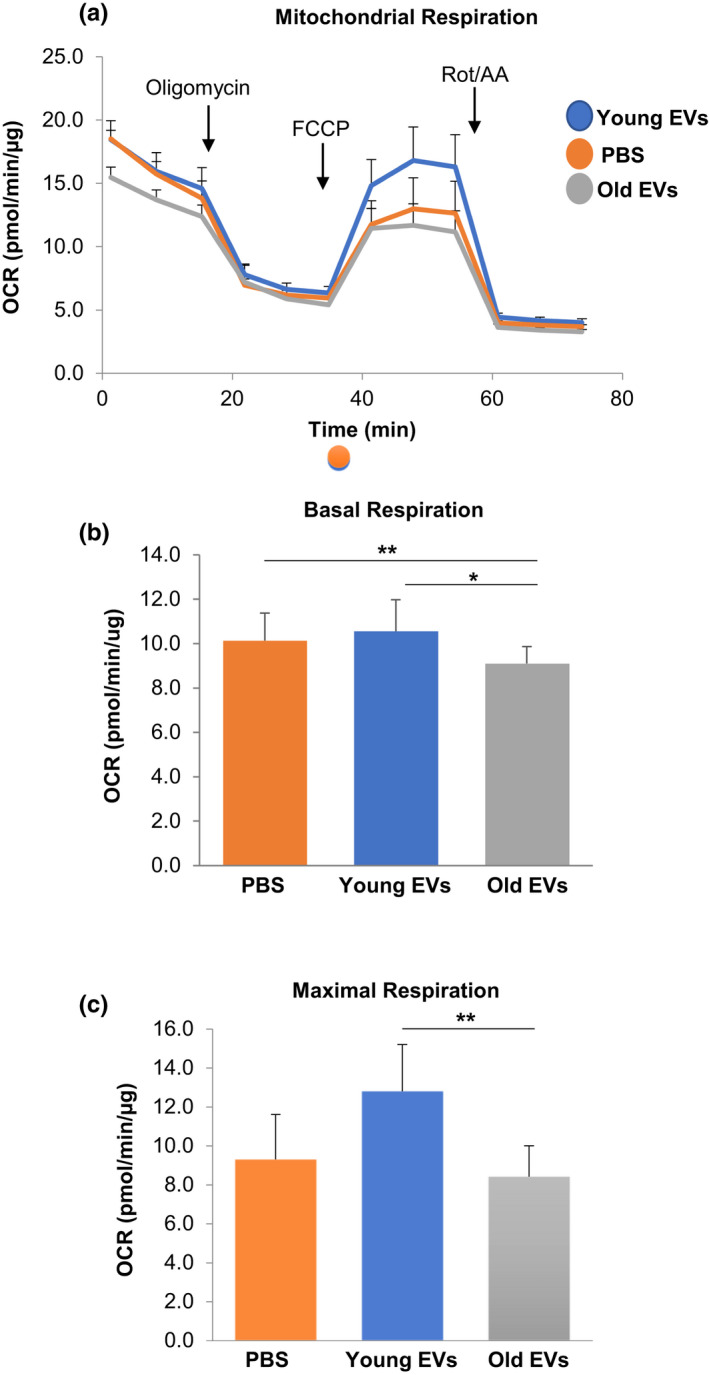
Plasma EVs alter mitochondrial function. HeLa cells were treated with EVs pooled from young and old individuals or PBS as a control for 16 h. (a) Oxygen consumption rate (OCR) was measured using the Seahorse XFE96 Analyzer. At the indicated times, oligomycin, FCCP, and Rot/AA were injected, and OCR values were obtained from the Agilent Mito Stress Test kit assay. (b) Basal respiration values were calculated from the Agilent Mito Stress Test kit assay. (c) Maximal respiration values were obtained from the Agilent Mito Stress Test kit assay. *p < 0.05, **p < 0.01 using a Student *t* test. Each treatment group is *n* = 10 replicates. Data shown represents mean + SEM. FCCP, Carbonyl cyanide‐4 (trifluoromethoxy) phenylhydrazone; AA, antimycin A

To determine whether age‐related changes in plasma EV mtDNA affect mitochondrial function, we pooled EV populations of young and old EVs and measured OCR. There were no significant differences in basal and maximal respiration between control (PBS treated) cells and cells treated with young EVs (Figure 6). Cells treated with young EVs had significantly higher levels of both basal and maximal respiration compared to those cells treated with old EVs (Figure 6). These data suggest that EVs affect mitochondrial energetics in an EV age‐dependent manner.

## 3 DISCUSSION

In this study, we found that ccf‐mtDNA in plasma EVs significantly declines with age in both our cross‐sectional and longitudinal analyses of a middle‐aged cohort of African Americans (AAs) and whites.

These findings may seem at first contradictory to a previous report finding that ccf‐mtDNA from plasma increased with age (Pinti et al., 2014). However, in that paper, they report a slight decline in ccf‐mtDNA levels from childhood (mean age = 6.8 years) to early adulthood (mean age = 33 years) and a gradual increase in ccf‐mtDNA levels in the ~60 years (mean age = 64 years) and elderly population (>90 years age). Our cohort at time 1 ranges from ~30–64 years and time 2 ~ 32–69 years. Therefore, we examined a largely middle‐aged cohort. In addition, our study includes AA and white individuals, whereas the Pinti study only includes white individuals. Also, of note, we have measured ccf‐mtDNA levels within EVs not just in whole plasma. Although we detected mtDNA in EVs, our data indicate that only a fraction of the total ccf‐mtDNA is contained in EVs. It is interesting since we detected very little ccf‐mtDNA in the EV‐depleted fraction during the EV isolation process. The first step in the plasma EV isolation process was the addition of thromboplastin D, which may indicate that ccf‐mtDNA may precipitate with the fibrin clot during this process or may be fragmented or not stable enough through the isolation procedure. Incorporating a DNase treatment of the EVs does indicate that the changes that we found with age are in mtDNA encapsulated in EVs. However, there may be differences in levels of ccf‐mtDNA in plasma and in EVs with age, which would be interesting to examine in future studies. Our data are consistent with previous reports that cellular mtDNA copy number decreases with age (Zhang et al., 2017) (Mengel‐From et al., 2014). Interestingly, in the Pinti study it was reported in a subset of those over 90 years. that higher inflammatory markers were associated with high mtDNA levels (Pinti et al., 2014). These data suggest that higher ccf‐mtDNA levels are associated with inflammation, termed “inflamm‐aging,” in the elderly (Franceschi et al., 2018). Other reports also suggest that ccf‐mtDNA levels are associated with inflammatory diseases including rheumatoid arthritis and HIV (Boyapati et al., 2017). Future work lies in investigating whether ccf‐mtDNA levels contribute to other inflammatory diseases.

Our study is unique because we examined ccf‐mtDNA contained in EVs from human plasma. In addition, we utilized 4 different primer sets that span various regions of the mitochondrial genome. This approach further validates our results and our technical reproducibility. Thus far, most studies have examined ccf‐mtDNA in serum or plasma (Boyapati et al., 2017) and used only one primer set (Lee et al., 2010; Pinti et al., 2014; Zachariah et al., 2008). We chose primer sets that were validated in the literature to amplify ccf‐mtDNA (Pinti et al., 2014; Shao et al., 2004; Walker et al., 2005), and we designed another unique primer set. In our cohort, the four different primer sets were highly correlated with each other and all showed a similar decline in mtDNA levels with age.

Limited studies have reported the presence of mtDNA in EVs. The majority of these reports have been from tissue culture cells (Guescini, Genedani, et al., 2010; Guescini, Guidolin, et al., 2010), but mtDNA could be detected in plasma EVs from women with hormonal therapy‐resistant breast cancer (Sansone et al., 2017). In this paper, mtDNA in EVs could be transferred to recipient cancer stem‐like cells and promote resistance to hormonal therapy. These data indicate that mtDNA present in EVs may be functional cargo. In agreement with this study, mesenchymal stem cell‐mediated transfer of mitochondrial components via EVs affects macrophage bioenergetics (Phinney et al., 2015). EVs containing mitochondria also convey inflammatory signals from activated monocytes to endothelial cells (Puhm et al., 2019). In a recent study, cell‐free intact potentially functional mitochondria could be isolated from plasma (Al Amir Dache et al., 2020). Here, we found that cells treated with EVs from young individuals had significantly higher levels of basal and maximal respiration compared to those cells treated with EVs from old individuals. These data suggest that aged EVs may impair mitochondrial function. The in vivo relevance of our data still needs to be investigated. However, this idea is in agreement with a recent report showing that injection of EVs from young mice into old mice increases mouse lifespan (Yoshida et al., 2019).

Cargo in EVs may relay cellular signals but may also be a mechanism for the removal of dysfunctional or damaged mitochondrial components (Picca et al., 2019). It has been reported that neurodegenerative‐related misfolded proteins are present in EVs (Janas et al., 2016). Autophagy has been shown to play a role in EV secretion (Freeman et al., 2018). It is well known that the removal of deficient mitochondria via mitophagy is altered with aging, along with pathways related to proteostasis (Lopez‐Otin et al., 2013). Therefore, it is interesting to speculate that the decline in mtDNA EV levels that we observe may reflect a defect in the clearance of cellular material with aging.

There are several limitations to our study. Here, we have examined individuals with an age range of ~30–64 at two different time points ~5 years apart. This approach allows us to perform two different cross‐sectional analyses and a longitudinal analysis. However, the age range of our cohort is younger than other studies examining ccf‐mtDNA with age (Pinti et al., 2014). In addition, this is a prospective study and there may be additional effects of freezing and thawing plasma on the stability of EV mtDNA levels. Previously we reported a relative stability in EV concentration for individuals between the two different visits, suggesting that freezing time does not significantly alter EV integrity (Eitan et al., 2017). Similar to other studies examining ccf‐mtDNA, we did not normalize our mtDNA levels to a reference gene since previously reported normalization genes did not amplify consistently in every sample and also the amplicon efficiencies of the mtDNA primer set and reference were not equal (Livak & Schmittgen, 2001). Given that the mtDNA primers were highly correlated to each other and not with EV concentration, we decided not to normalize the data to EV concentration. Although our visualization of qPCR products indicated a single amplification product for our mtDNA primer sets, we cannot exclude that more mtDNA fragmentation occurs with age and this may contribute to the decreased mtDNA levels observed.

In conclusion, we report a decline in EV‐associated mtDNA levels with human age. Given the striking mid‐life mortality rates, it is important to identify novel biomarkers that may be indicators of health status not only in the elderly but also in middle‐aged populations (Xu et al., 2020). Previous data suggest that EV concentration and protein cargo are associated with traditional clinical markers of mortality (Noren Hooten et al., 2019). Our data shed new light onto how EV cargo changes in humans with age and indicate that EV‐associated mtDNA has the potential to be utilized as a clinical biomarker of aging.

## 4 EXPERIMENTAL PROCEDURES

### 4.1 Clinical study participants

A subcohort of young (30–35 years), middle‐aged (40–55 years), and old individuals (55–64 years) was selected from the Healthy Aging in Neighborhoods of Diversity across the Life Span (HANDLS) study of the National Institute on Aging Intramural Research Program, National Institutes of Health (NIH) (Evans et al., 2010). HANDLS is approved by the Institutional Review Board of the National Institutes of Health. All participants provided written informed consent. HANDLS is a longitudinal study comprised of community‐dwelling participants and aims to investigate the role of race and socioeconomic status on the development of age‐associated health disparities. Previously, we analyzed EVs in a larger cohort (*n* = 74) of individuals at two different time points ~5 years apart (4.60 ± 1.04 years) (Eitan et al., 2017). Here, we examined a subset of these participants from whom we had available isolated EVs (*n* = 55 at visit 1 and *n* = 67 at visit 2). The cohort contained both African American (AA) and white participants. Plasma samples were collected after overnight fasting. Participants that were diagnosed with cancer, Alzheimer's disease, HIV or who were Hepatitis B surface antigen or Hepatitis C antibody positive were excluded from the cohort. Participant demographics are detailed in Table 1.

Body mass index (BMI) was calculated from participant height and weight (BMI = Weight [kg]/Height [m^2^]). Measurements and clinical data were obtained during a structured medical history interview and a physical examination.

### 4.2 Plasma EV Isolation

For the experiments in this study, we utilized previously isolated plasma derived total EVs (Eitan et al., 2017). Briefly, plasma EVs were isolated from 0.45 ml plasma using ExoQuick™ Exosome Precipitation Solution (System Bioscience Inc.) as described (Eitan et al., 2017). This isolation method was used since it provided more reproducible results for EV isolation from large human cohorts (Eitan et al., 2017). Isolated EVs were stored at −80°C prior to their use for this project.

For the pooled plasma experiments, four individual plasma samples were pooled together and then separated into 0.5 ml aliquots. This was done two separate times, each time using plasma from a different set of four individuals. This created two independent pooled populations of plasma to undergo EV isolations. The 0.5 ml pooled plasma aliquots were treated with 0.2 ml Thromboplastin D (Cat#:100354, Fisher Scientific, Inc.) and incubated at room temperature for 30 min. Then, 0.3 ml of Dulbecco's phosphate‐buffered saline (DPBS^−2^) with 3× concentrated protease and phosphatase inhibitor cocktails (Roche Applied Sciences) was added. The samples were centrifuged at 3000 *g* for 20 min at 4°C. Subsequently, 252 µl of ExoQuick™ Exosome Precipitation Solution (System Bioscience Inc.) was added per sample, incubated for 1 h at 4°C, and then centrifuged at 1500 *g* at 4°C for 20 min. The supernatant was separated and saved for analysis as the EV‐depleted plasma fraction. The EV pellet was resuspended in 0.5 ml of DPBS^−2^ with 3× concentrated protease and phosphatase inhibitor cocktails (Roche Applied Sciences).

For Figure 2c, plasma from the same individuals as above were pooled creating two different pooled plasma sets. Plasma (50 µl) was aliquoted into 3 tubes, and plasma (500 µl) was aliquoted into 3 tubes for EV isolations using ExoQuick™. These volumes were utilized since 50 µl of the 500 µl EV pellet was used for the DNA isolations. EV‐depleted fractions were also collected as described above.

For the EV isolation comparison experiments, plasma samples (*n* = 8) were pooled together and separated out into eight 0.5 ml aliquots. Four aliquots were isolated using ExoQuick™ as described above. The other four 0.5 ml aliquots were equalized to 5 ml using sterile PBS. The samples were centrifuged at 500 *g* for 10 min at 4°C. The supernatant was placed into a fresh tube and centrifuged at 2500 *g* for 10 min at 4°C. The supernatant was then transferred to ultracentrifugation tubes, and the samples were centrifuged at 120,000 *g* for 2 h at 4°C in a Beckman Coulter Optima XPN‐100 Ultracentrifuge (SW 55 Ti rotor, *K* = 48). The supernatant collected from this spin was considered the “EV‐depleted” fraction. The resuspended pellet was then centrifuged again at 120,000 *g* for 2 h at 4°C. The supernatant was discarded, and the pellet remaining was considered our EV fraction, which was resuspended in 100 µl of PBS.

### 4.3 Nanoparticle tracking analysis

Isolated EV samples from two independent populations of pooled plasma samples were diluted 1:300 in filtered PBS. Vesicle size distribution and concentration was analyzed using nanoparticle tracking analysis (NTA) on a NanoSight NS500 (Malvern Instruments Ltd.). Samples were recorded at camera level = 14, detection level = 3, and 5 videos of 20 s with a coefficient of variance <10% were used for analysis. Software NTA 3.3 Dev Build 3.3.104 was used. All samples were analyzed on the same instrument by a single operator. For the cohort participant samples, the EV concentrations and size distributions were previously measured and described (Eitan et al., 2017).

### 4.4 Transmission electron microscopy

The Electron Microscopy imaging was done by the Johns Hopkins University Neurology Microscopy Core as previously described (Eitan et al., 2017; Freeman et al., 2018). Briefly, the grids were visualized on a Libra 120 Transmission Electron Microscope at 120 kV (Zeiss). The images were taken with a Veleta camera (Olympus).

### 4.5 Immunoblotting

Four EV samples from one of the aliquoted pooled plasma populations were lysed in Mammalian Protein Extraction Reagent (MPER™). These EV samples, HeLa cell lysate, and four plasma EV‐depleted supernatant samples from the same pooled plasma samples were subjected to SDS‐PAGE and immunoblotted. Protein samples of 10 μg were loaded, separated on 4%–12% NuPAGE Bis‐Tris gels under SDS‐denaturing conditions (Invitrogen Life Technologies, Grand Island, NY), and transferred onto a nitrocellulose membrane. The membrane was incubated with primary antibodies at 1:500 dilutions for 1 h at room temperature: CD81 (System Biosciences clone EXOAB‐CD81A‐1 kit), FLOT1 (Abcam clone EPR6041), and GM130 (Abcam clone EP892Y). For detection, the membranes were incubated with the appropriate secondary horseradish peroxidase (HRP)‐conjugated antibody. 1:20,000 dilutions of the appropriate secondary horseradish peroxidase (HRP)‐conjugated antibody (System Biosciences, EXOABCD81A‐1) for 40 minutes at room temperature was used for CD81 and 1:5,000 dilutions of the appropriate secondary horseradish peroxidase (HRP)‐conjugated antibody (GE Healthcare UK Limited, NA934V) for 40 min at room temperature were used for FLOT1 and GM130. The blots were visualized using Pierce ECL Plus Western Blotting Substrate according to manufacturer's protocol (Catalog number: 32132).

### 4.6 DNA isolation from plasma‐derived EVs

Plasma EV samples (50 µl), previously isolated and stored at −80°C (Eitan et al., 2017), were DNase treated (Lucigen, Cat: DB0715K) in order to degrade any DNA material on the outside of the EVs. For experiments comparing DNase treatment to those without, the DNase was omitted, and reactions were carried out in parallel. EVs were treated using 6.5 µl of DNase Reaction Buffer, and 5 U of DNase per reaction at 37°C for 30 minutes. The reaction was halted by the addition of 6.5 µl DNase Stop Solution per reaction at 65°C for 10 minutes. Each sample was then equalized to 200 µl with nuclease free water and lysed through the addition of 20 µl Proteinase k included in the DNeasy Blood and Tissue kit (Qiagen, Cat: 69506). The EV‐depleted samples (200 µl) were lysed through the addition of 20 µl Proteinase k included in the DNeasy Blood and Tissue kit. From there, the standard manufacturer's DNA extraction protocol was followed with the following exceptions. The “optional” spin at 20,000 *g* for 1 min after AW2 addition was included, and waste collection tubes were changed in between each spin. In addition, the final 1 min at 8,000 *g* spin for DNA elution was prefaced by a 5‐min incubation at room temperature of 50 µl AE Buffer in the spin column. The eluted DNA (~50 µl) was diluted in another 50 µl of AE Buffer (Qiagen) and stored at −20°C prior to a qPCR assay.

### 4.7 Quantitative real‐time PCR

For quantitative real‐time PCR (qPCR) analysis, each of our participant samples was run blinded to participant information, and the two collection points were analyzed at the same time, for each primer set, to minimize variability. Samples were run in duplicate and the duplicate mean was used in subsequent analysis. qPCR was performed using mitochondrial gene‐specific primers (2.5 µl/rxn), TaqMan™ Fast Advanced Master Mix (7.5 µl/rxn) and 3 µl of DNA per reaction, for a total of 13 µl reactions. Primer sequences are listed in Table S1. We designed the Mito_3164 primer set and the Mito_4625, Mito_7878 and Mito_8446 primer sets were previously reported (Pinti et al., 2014; Shao et al., 2004; Walker et al., 2005). A 7900HT Fast Real‐Time PCR System was used to run the samples (Applied Biosystems). The thermal profile for mtDNA qPCR was as follows: 3 min at 95 degrees Celsius followed by 40 cycles of 20 s at 95 degrees Celsius and 20 s at 60 degrees Celsius. The relative expression of each mtDNA primer set was calculated using a derivation of the 2^−ΔΔCt^ method and normalized on the global mean (X; mean of the Ct for each primer set). Therefore, the following formula was used 2^−(X−Ct)^. We used this derivation of the 2^−ΔΔCt^ method since a reliable internal control could not be obtained using DNA isolated from plasma EVs. We attempted to amplify several different previously reported genes for normalization, but these genes did not amplify consistently in every sample and also the amplicon efficiencies of the target and reference were not equal as suggested for using a proper reference value (Livak & Schmittgen, 2001).

For the comparison between isolation techniques, mtDNA levels were normalized to the volume of EV input 50 µl /100 µl f.v in UC versus 50 µl /500 µl f.v. for ExoQuick™. In the histograms, mtDNA levels were normalized to the EV group for each different primer set. For the EV mtDNA levels with age analysis, the values were positively skewed and thus were log_2_ transformed.

### 4.8 Mitochondrial activity assay

HeLa cells were grown in 10% FBS in DMEM. HeLa cells were switched to EV‐free FBS (10%; Gibco) in DMEM and were seeded (4E4 cells/well) onto 96‐well Seahorse cell culture microplates (Agilent, part no. 101085‐004). After 24 h, pooled EVs from either young participants (ages 30.1, 30.2 years) or old participants (ages 67.8, 69.6 years) were added at a dose of 8.1E4 EVs/well (Eitan et al., 2017). Small volumes were utilized for EV doses for young (0.28 µl) and old EVs (0.30 µl) to minimize effects on the assay, and volumes were all equalized to 36 µl (20% of final media volume) with PBS. The cells were incubated with the EVs for 16 h. Desired dose was obtained after testing 3E6, 3E5, and 8.1E4 pooled EVs/well (Figure S3). The EV pool used for dose testing was representative of mixed age groups (*n* = 4). PBS was used as a control. Flux pack sensors (Agilent, part no. 102416‐100) were hydrated in sterile water overnight at 37 °C in a non‐CO_2_ incubator, prior to being immersed in XFe calibrant solution (Agilent, part no. 100840‐000) 1 h before machine calibration. HeLa cells were then incubated in Seahorse base medium (Agilent, part no. 102353‐100) containing 10% EV‐free FBS, glutamine (1 mM), glucose (10 mM), and sodium pyruvate (1 mM). The pH of the media was adjusted to 7.4. Oxygen consumption rate (OCR) was measured with the Seahorse Mito Stress Test Assay, utilizing the manufacturer's protocol (Agilent Technologies). During the mitochondrial stress test, cells were treated with oligomycin (1.5 mM), carbonyl cyanide‐4 (trifluoromethoxy) phenylhydrazone (FCCP) (1 µM), and rotenone/antimycin A (0.5 mM), at 14.29, 33.8, and 53.35 minutes, respectively. Oxygen consumption rates (OCR) were recorded in real time for mitochondrial stress tests using a Seahorse XFe96*^®^* analyzer (Agilent). All parameters were normalized to protein concentration per well (BCA assay) and calculated using Wave 2.4.0 software.

### 4.9 Statistics

Statistical analysis was performed using R software version: R 3.6 (R Core Team, 2019). One‐way ANOVA was used to test the differences among the age groups for BMI and age and Pearson's chi‐squared test was used for race and sex. Variables with positively skewed distributions were log_2_ transformed as indicated. Correlations between primers and with EV concentration were assessed by Pearson correlation coefficients (*r*) with degrees of freedom accounting for the matching by race and sex. Linear mixed models, accounting for matching by sex and race and repeated measurements, were used to analyze the cohort both longitudinally and cross‐sectionally. Significance of fixed factors was determined by log likelihood tests.

## CONFLICT OF INTEREST

The authors declare that they have no conflicts of interest.

## AUTHOR CONTRIBUTIONS

NNH, SL, and EE conceived and designed the study with help from MKE. SL, NNH, and JG executed the experiments. NM and ABZ performed statistical analysis. PG and QL helped with technique development for the study. ABZ and MKE are co‐principal investigators for HANDLS. Research was conducted in the laboratories of MKE and MPM. SL and NNH wrote the manuscript with input from all the authors. MKE is the guarantor of this work and, as such, had full access to all the data in the study and takes responsibility for the integrity of the data and the accuracy of the data analysis.

## Supporting information

Supplementary Material

## Data Availability

The datasets generated and analyzed during the current study are available from the corresponding author on reasonable request through the HANDLS website https://handls.nih.gov/.
