# Supplementary material for: Mitochondrial DNA in extracellular vesicles declines with age
Source: Aging Cell. 2020 Dec 23;20(1):e13283. doi: 10.1111/acel.13283 (PMC7811845; doi:10.1111/acel.13283)
Supplement: Supplementary file 1 — Supplementary Material [file ACEL-20-e13283-s001.pdf]

Supporting Information

Mitochondrial DNA in Extracellular Vesicles Declines with Age

Stephanie Lazo[1*,] Nicole Noren Hooten[1*,] Jamal Green[1,4,] Erez Eitan[2,5,] Nicolle A. Mode[1,] Qing-Rong Liu[3,] Alan B. Zonderman[1,] Ngozi Ezike[1,] Mark P. Mattson[2,] Paritosh Ghosh[3,] and Michele K. Evans[1,#]

[1]Laboratory of Epidemiology and Population Science, [2]Laboratory of Neuroscience, [3]Laboratory of Clinical Investigation National Institute on Aging, National Institutes of Health, 251 Bayview Boulevard, Baltimore, MD 21224, [4] Present address: University of Pennsylvania, Perelman School of Medicine, Philadelphia, PA 19104, Present address: [5]NeuroDex, Natick, MA

[*]These authors contributed equally to this work.

Supporting Information Table S1. Primer Sequences for qPCR

Gene	Name	Sequence	Reference	Size (bp)
MT-RNR2/MT-TL1	Mito_3164 Forward	5'CCTTCCCCCGTAAATGATATCA3'	unpublished	76
MT-RNR2/MT-TL1	Mito_3164 Reverse	5'GCCATCTTAACAAACCCTGTTCTT3'	unpublished	
MT-RNR2/MT-TL1	Mito_3164 Probe	5'FAM-AACTTAGTATTATACCCACACCC-MGB3'	unpublished	
MT-ND2	Mito_4625 Forward	5'CACAGAAGCTGCCATCAAGTA3'	Pinti et al., Eur. J. Immunol. 2014	89
MT-ND2	Mito_4625 Reverse	5'CCGGAGAGTATATTGTTGAAGAG3'	Pinti et al., Eur. J. Immunol. 2014	
MT-ND2	Mito_4625 Probe	5'FAM-CCTCACGCAAGCAACCGCATCC-BLACK HOLE-3'	Pinti et al., Eur. J. Immunol. 2014	
MT-COX2	Mito_7878 Forward	5´AATCAATTGGCGACCAATGG3´	Shao et al., World Journal Gastroenterology, 2004	100
MT-COX2	Mito_7878 Reverse	5´CGCCTGGTTCTAGGAATAATGG3´	Shao et al., World Journal Gastroenterology, 2004	
MT-COX2	Mito_7878 Probe	5'FAM-ACTGAACCTACGAGTACAC-MGB-3'	Shao et al., World Journal Gastroenterology, 2004	
MT-ATP8	Mito_8446 Forward	5'AATATTAAACACAAACTACCACCTACCT3'	Walker et al., Journal Analytical Biochemistry 2005	79
MT-ATP8	Mito_8446 Reverse	5'TGGTTCTCAGGGTTTGTTATAA3'	Walker et al., Journal Analytical Biochemistry 2005	
MT-ATP8	Mito_8446 Probe	5'-FAM-CCTCACCAAAGCCCATA-MGB-3'	Walker et al., Journal Analytical Biochemistry 2005	

References

Pinti, M., Cevenini, E., Nasi, M., De Biasi, S., Salvioli, S., Monti, D., . . . Cossarizza, A. (2014). Circulating mitochondrial DNA increases with age and is a familiar trait: Implications for "inflamm-aging". *Eur J Immunol, 44*(5), 1552-1562. doi:10.1002/eji.201343921

Shao, J. Y., Gao, H. Y., Li, Y. H., Zhang, Y., Lu, Y. Y., & Zeng, Y. X. (2004). Quantitative detection of common deletion of mitochondrial DNA in hepatocellular carcinoma and hepatocellular nodular hyperplasia. *World J Gastroenterol, 10*(11), 1560-1564. doi:10.3748/wjg.v10.i11.1560

Walker, J. A., Hedges, D. J., Perodeau, B. P., Landry, K. E., Stoilova, N., Laborde, M. E., . . . Batzer, M. A. (2005). Multiplex polymerase chain reaction for simultaneous quantitation of human nuclear, mitochondrial, and male Y-chromosome DNA: application in human identification. *Anal Biochem, 337*(1), 89-97. doi:10.1016/j.ab.2004.09.036

Supp. Figure S1

a

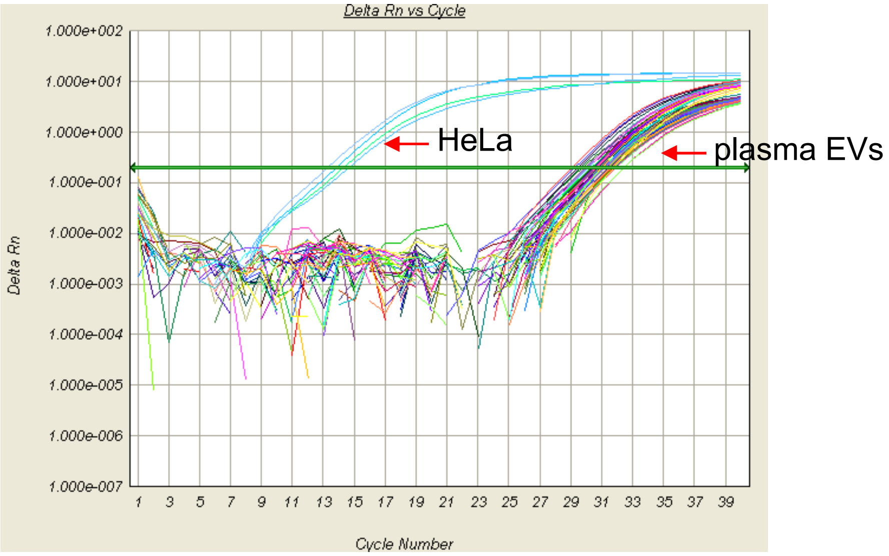

b

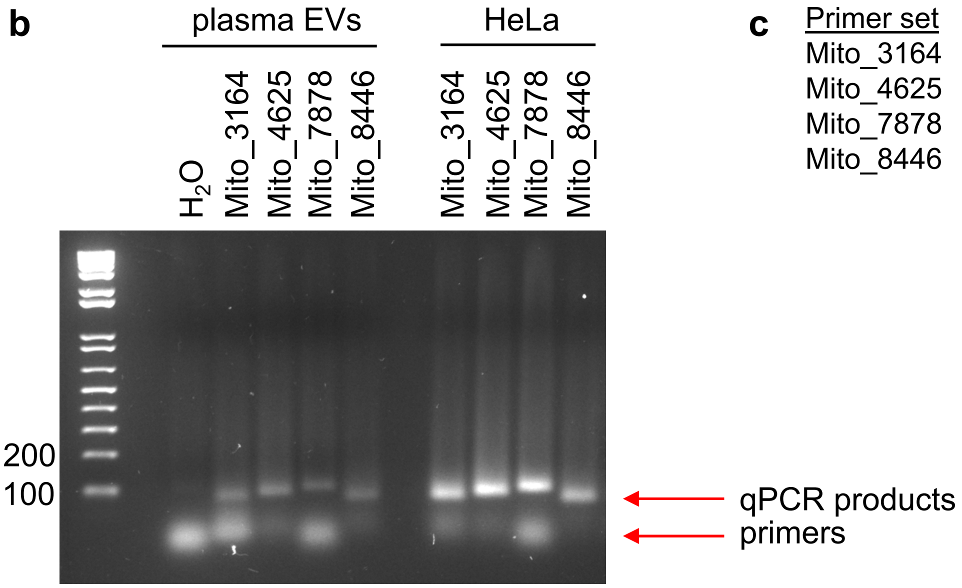

c

Primer set	Size
Mito_3164	76 bp
Mito_4625	89 bp
Mito_7878	100 bp
Mito_8446	79 bp

Supporting Information Figure S1. mtDNA qPCR analysis. a) EVs were isolated from pooled plasma and then DNA was isolated from either the plasma EVs or from HeLa cells as a positive control. mtDNA levels were measured using mtDNA specific primers from four different regions of the mitochondrial genome using qPCR. qPCR amplification plots are shown in (a). The qPCR products from these reactions were visualized by electrophoresis on SYBR-safe (Invitrogen) stained gels. DNA gels were used to visualize that a single amplification product was observed in the reactions. (c) Expected size of the amplicon for each qPCR product for each primer set is listed.

Supp. Figure S2

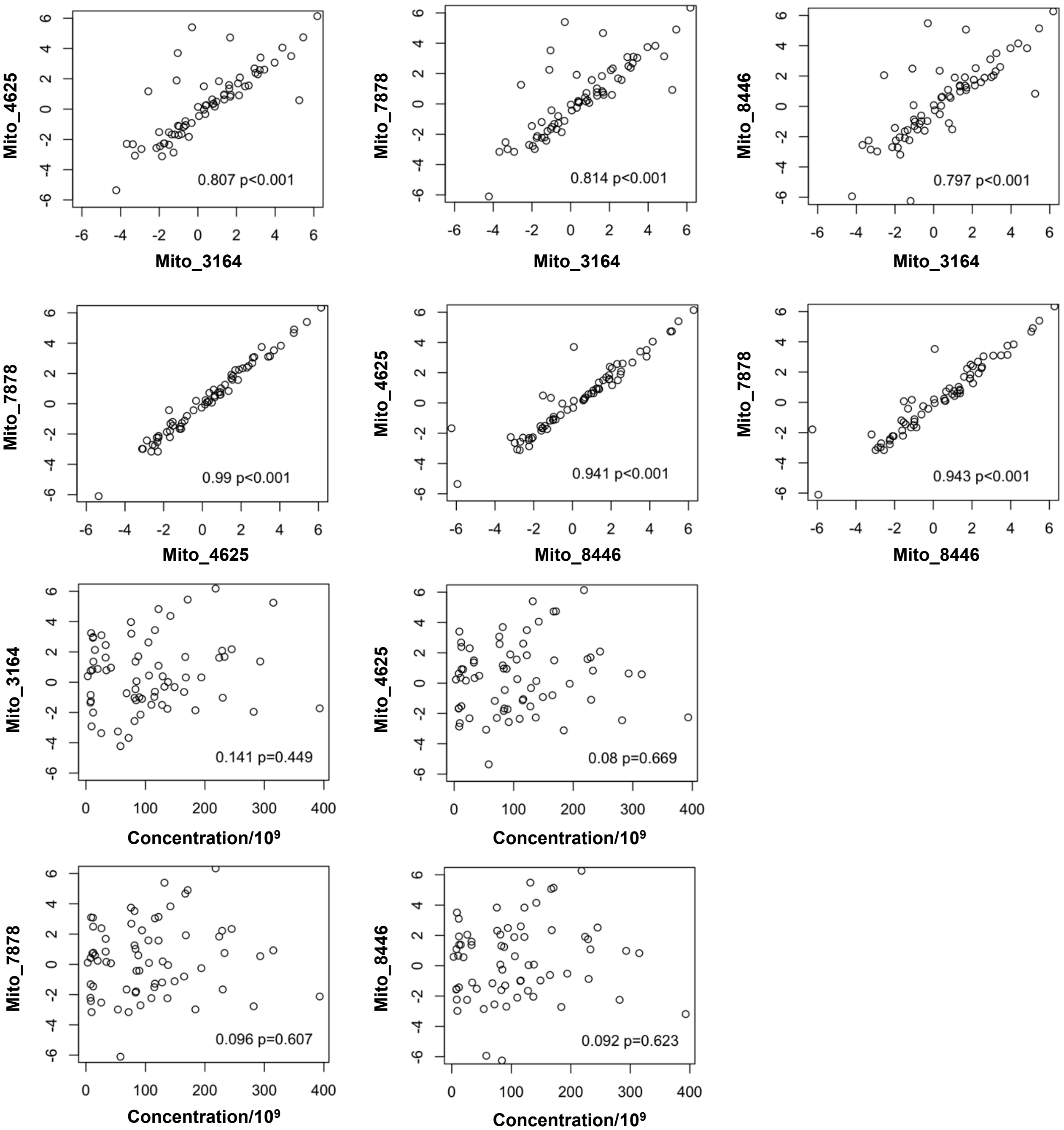

Supporting Information Figure S2. mtDNA primers positively correlate with each other but not with EV concentration. a) EVs were isolated from plasma from 67 individuals across different age groups at visit 2. DNA was isolated and circulating cell-free mtDNA levels were measured using mtDNA specific primers from four different regions of the mitochondrial genome via qPCR. Correlations between primers and between primer and EV concentration were assessed by Pearson correlation coefficients (r) with degrees of freedom accounting for the matching by race and sex. r values and P values are indicated. Similar results were obtained for visit 1 (Figure 3). b) EV concentration was obtained using Nanoparticle Tracking Analysis and the relationship between EV concentration and EV mtDNA levels were analyzed using linear mixed model regression. r values and P values are indicated. Similar results were obtained for visit 1 (Figure 3).

Supp. Figure S3

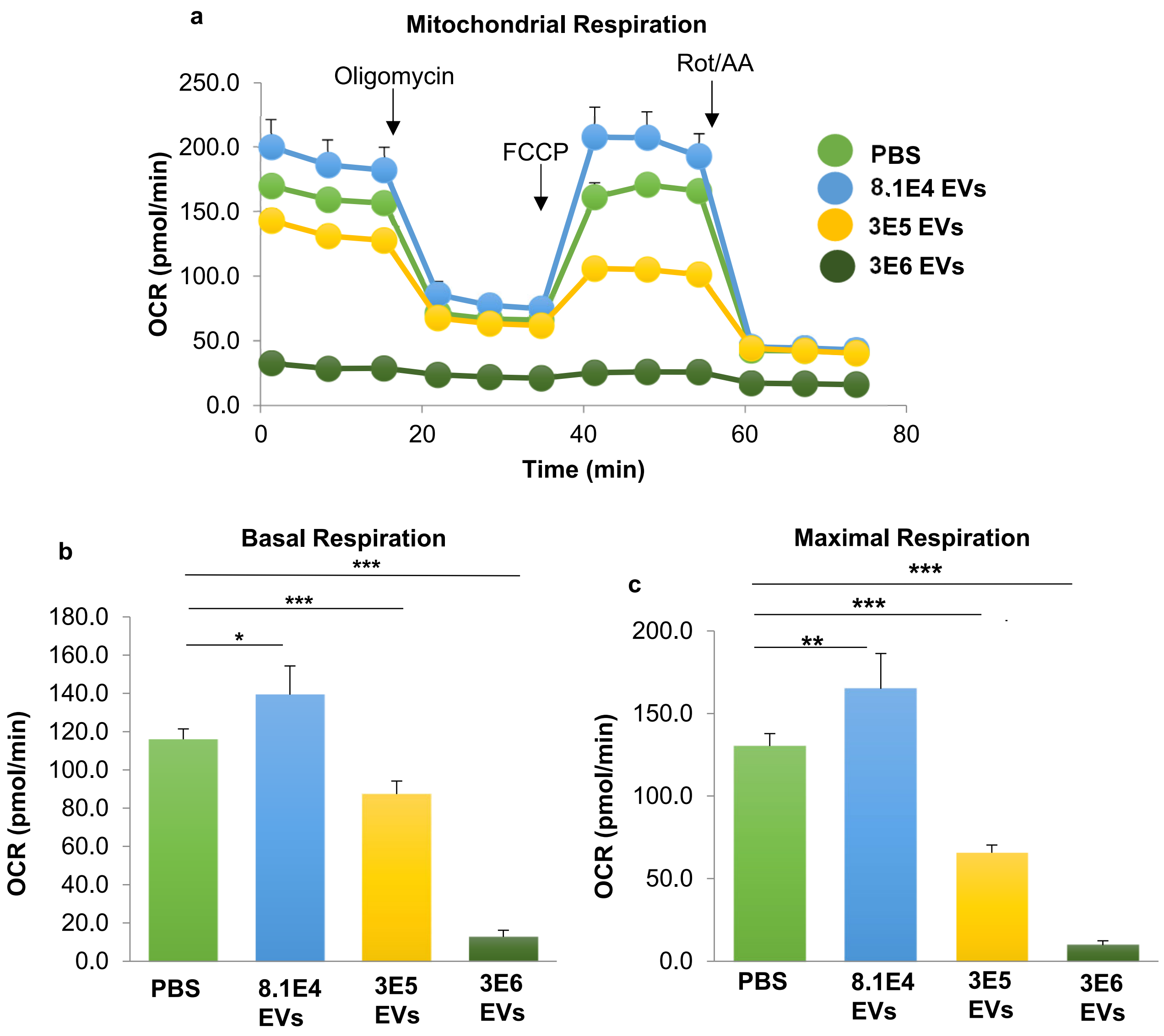

Supporting Information Figure S3. Plasma EV dose affects mitochondrial function. HeLa cells were treated with EVs pooled from 4 individuals or PBS as a control for 16 hrs. a) Oxygen consumption rate (OCR) measured with the Seahorse XFE96 Analyzer. At the indicated times, oligomycin, FCCP, and Rot/AA were injected, and OCR values were calculated from the Agilent Mito Stress Test kit assay. b) Basal respiration values were calculated from the Agilent Mito Stress Test kit assay. c) Maximal respiration values were calculated from the Agilent Mito Stress Test kit assay, *$P < 0.05$, **P<0.01, ***$P < 0.001$. Data shown represents the mean (n=10) + SEM.
